# Drug delivery for fighting infectious diseases: a global perspective

**DOI:** 10.1007/s13346-021-01009-1

**Published:** 2021-06-09

**Authors:** Brigitta Loretz, Yu-Kyoung Oh, Sarah Hudson, Zhen Gu, Claus-Michael Lehr

**Affiliations:** 1 Helmholtz Institute for Pharmaceutical Research Saarland (HIPS), Helmholtz Center for Infection Research (HZI), Saarbrücken, Germany; 2 College of Pharmacy and Research Institute of Pharmaceutical Sciences, Seoul National University, Seoul, 08826 Republic of Korea; 3 Department of Chemical Sciences, SSPC the Science Foundation Ireland Research Centre for Pharmaceuticals, Bernal Institute, University of Limerick, Limerick, Ireland; 4 College of Pharmaceutical Sciences, Zhejiang University, Hangzhou, 310058 China; 5 Department of General Surgery, School of Medicine, Sir Run Run Shaw Hospital, Zhejiang University, Hangzhou, 310016 China; 6 Zhejiang Laboratory of Systems & Precision Medicine, Zhejiang University Medical Center, Hangzhou, 311121 China; 7 MOE Key Laboratory of Macromolecular Synthesis and Functionalization, Department of Polymer Science and Engineering, Zhejiang University, Hangzhou, 310027 China; 8 Department of Pharmacy, Saarland University, Saarbrücken, Germany

Epidemiologists have predicted that viral infections might spread fast in our highly globalized world. For example, a report on “Preparedness for a high-impact respiratory pathogen pandemic” was published in September 2019 by Johns Hopkins Centre for Health Security [1]. This report ranked aerosol transmitted viral diseases as the highest risk for a wide-spreading pandemic and named, as an example beyond influenza, also severe acute respiratory syndrome (SARS) as a zoonotic coronavirus. In approximately 1 year, SARS-CoV-2 (coronavirus type 2) has developed into a true pandemic and reminds us that problems must be taken seriously in a timely manner. In the case of the current coronavirus disease 2019 (COVID-19), we profited from the protocols established during earlier pandemics. Following the influenza (H1N1) pandemic in 2009, a pandemic preparedness plan was developed by the World Health Organization (WHO). SARS-CoV infections in 2003 and MERS (Middle East Respiratory Syndrome) in 2012 lead to research in this type of Coronaviridae and strategies to combat them. The Ebola epidemic in West Africa 2014–2016 activated a push for programs in preventive and therapeutic strategies driven by governmental and non-governmental institutions. We now profit from many of these efforts by building on SARS biological and medical research anti-viral compound candidates and the fastest ever approved vaccine developments in history so far. A lesson to learn from this pandemic could be that preparedness may reduce the impact of disasters, and spending money on contingency measures may be well invested.

**Fig. 1:**
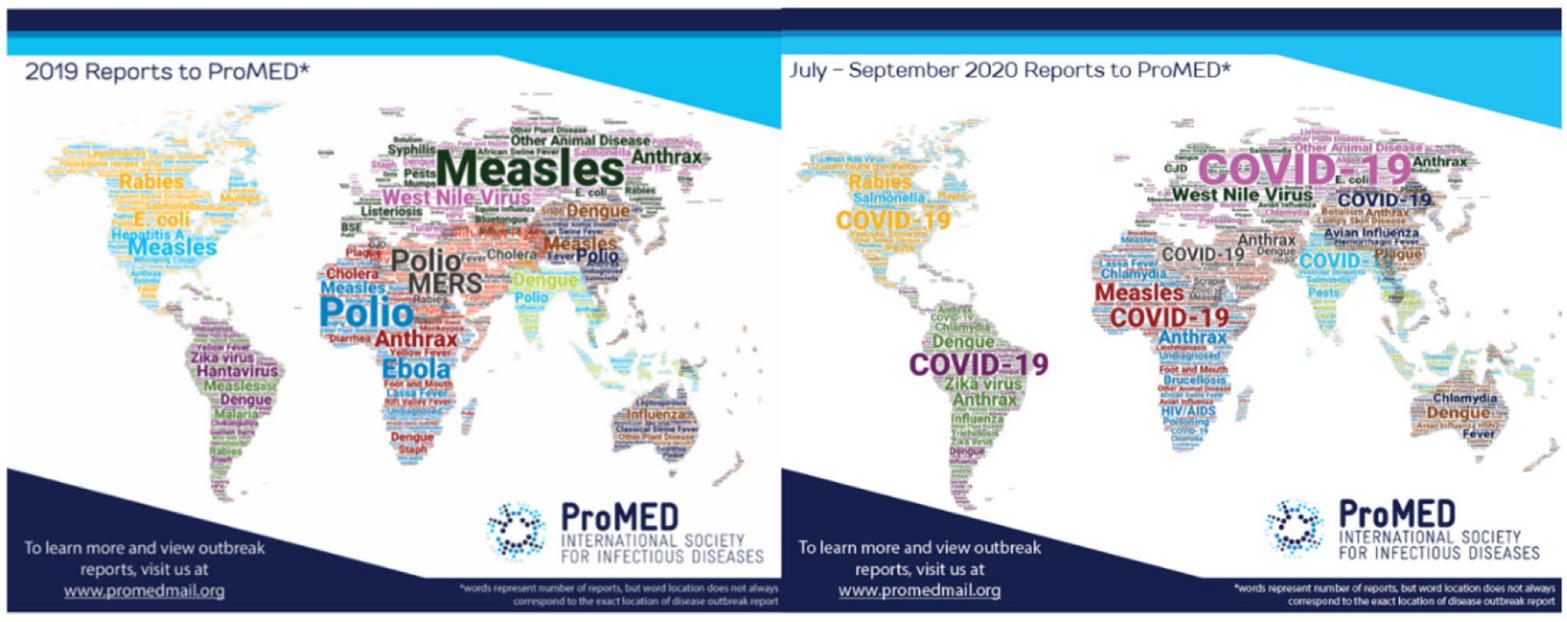
Word cloud published by the International Society of Infectious Diseases (ISID) for the reported disease outbreaks in the year 2019 (left) and quarter three of 2020 (right). Word size is an indication of the frequency of ProMED reports. ISID provides global surveillance of infectious diseases (https://isid.org/surveillance/). Despite the prominent role of COVID-19 in 2020, there is still a multitude of other infectious disease outbreaks

For the moment, viral infections are present in our minds. However, the awareness the COVID-19 pandemic has created for infectious diseases should go beyond the current threat of this new coronavirus. Even today, COVID-19 is the most prominent but by far not the only actual infectious disease outbreak (Fig. 1).

The coevolution of potentially pathogenic organisms with humans and our environment sets the basis for a continuing battle. A list of human pathogens, reaching a total of 2107, was gathered in 2008 (ranked by the number of species: approximately 1000 bacteria, 440 fungi, 300 helminths, 270 viruses, and 80 protozoa) [2]. Certain regions in the world have a higher burden of zoonotic infectious disease occurrence, originating from their higher biodiversity [3]. Unfortunately, in some regions, this risk is correlated with limited resources to prepare against such threats. Most pathogens have a low spreading tendency and often, for vector or climate reasons, become endemic only in some regions of the world. However, changes in natural habitats, climate change, and human mobility affect the global distribution of pathogens. Infectious diseases are a global threat and challenge, which should be tackled promptly and in unison across the world. The sporadic emergence of new human pathogens shifts in geographic distributions, and on top of this, the development of antimicrobial (antibiotics, antiviral, antifungal, and antiparasitic actives) resistance emphasizes the urgent need to maintain and intensify our efforts in anti-infective research to ensure as much preparedness as possible. Not only the appearance of new human pathogens is an issue but climate change can contribute to increased incidence or shifts in geographical distribution (for example, Zika or Dengue viruses transmitted by mosquitoes, or *Borrelia burgdorferi* by ticks). In today’s world, infectious diseases are a leading cause of death worldwide, particularly in low- to middle-income countries, and especially in young children. Lower respiratory tract infections (e.g., pneumonia, also including chronic obstructive pulmonary disease (COPD) and influenza) are still in the top-ten killer diseases worldwide, particularly high in ranking in low-income countries accompanied in that list by diarrheal diseases, malaria, and tuberculosis (Table 1) [4]. The number of deaths from lower respiratory infections declined between 2000 and 2019, as well as the impact of diarrheal diseases, tuberculosis, and HIV/AIDS. However, because of rising antimicrobial resistance, the COVID-19 pandemic and economic crises, the threat of a reversal in this decline in death rate from such infectious diseases is imminent.

**Table 1:** The top ten leading causes of death ( source: WHO Report 2020). Infectious diseases are highlighted in bold

Rank	Low-income countries	High-income countries	Worldwide
1	Neonatal conditions	Ischemic heart disease	Ischemic heart disease
2	**Lower respiratory infections**	Alzheimer’s disease	Stroke
3	Heart disease	Stroke	Chronic obstructive pulmonary disease (COPD)
4	Stroke	Lung cancers	**Lower respiratory infections**
5	**Diarrheal diseases**	COPD	Neonatal conditions
6	**Malaria**	**Lower respiratory infections**	Lung cancers
7	Road injury	Colon cancers	Alzheimer’s disease and dementias
8	**Tuberculosis**	Kidney diseases	**Diarrheal diseases**
9	**HIV/AIDS**	Hypertensive heart disease	Diabetes
10	Cirrhosis of the liver	Diabetes	Kidney diseases

Our medications against pathogens are losing their efficacy for various species. Tuberculosis and malaria are major killer diseases with severe resistance problems. Importantly, several bacterial human pathogens (especially the six highly virulent pathogens *Enterococcus faecium*, *Staphylococcus aureus*, *Klebsiella pneumoniae*, *Acinetobacter baumannii*, *Pseudomonas aeruginosa*, and *Enterobacter* species, a.k.a. ESKAPE) show a rising tendency of antimicrobial resistance (AMR) and spread not only in the hospital environment but also through community transmission. An estimated 700,000 deaths worldwide, thereof alone 230,000 from multidrug-resistant (MDR) tuberculosis, are claimed by AMR today [5]. Drug-resistant microbes endanger populations with poor sanitation as well as surgical inventions in developed countries’ clinics. Therefore, worldwide calls have been issued to develop new anti-infectives and use the existing ones with more caution. One hundred and twenty-seven countries have developed a national action plan to tackle AMR [6].

**Fig. 2:**
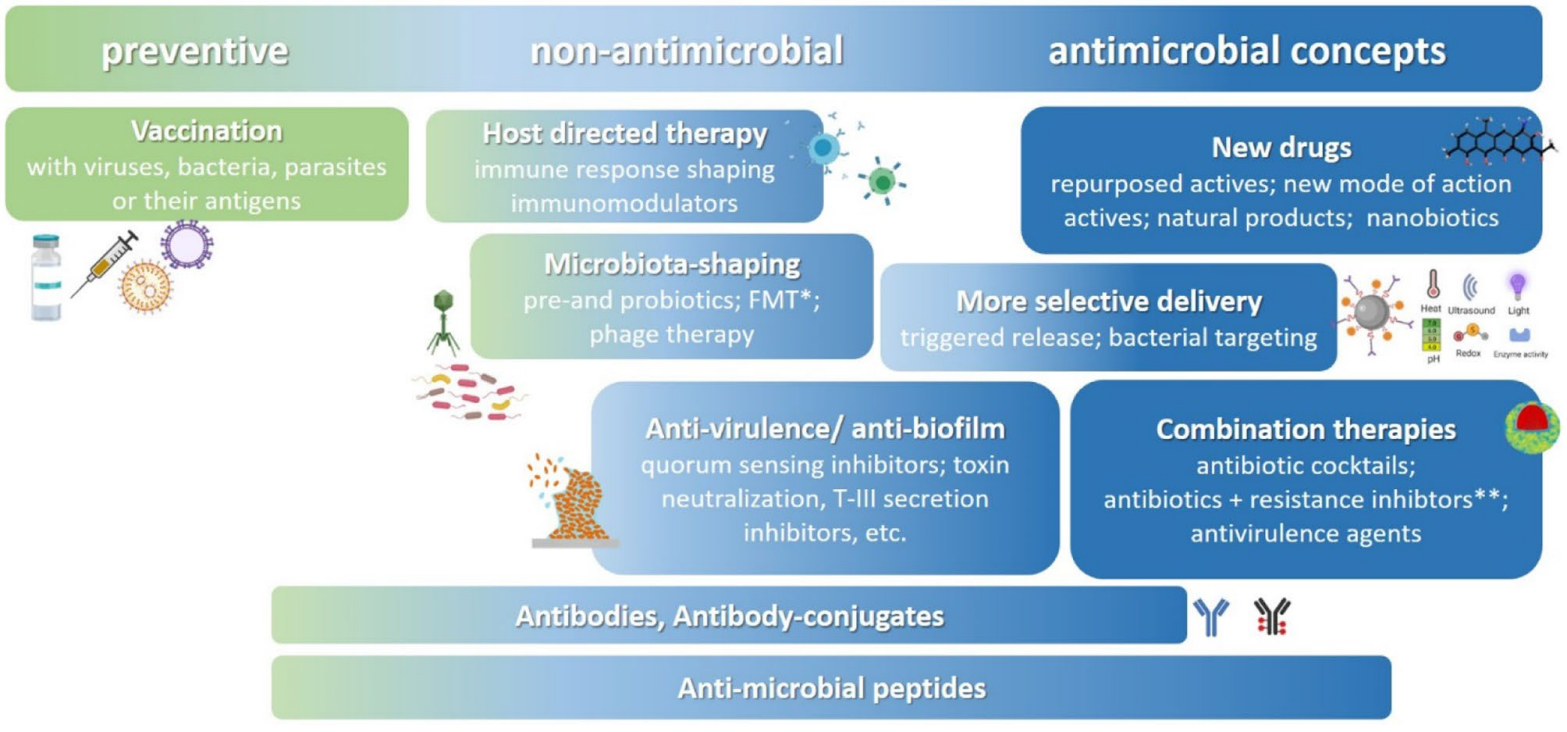
Anti-infective strategies are getting more diverse, comprising many concepts in development pipelines. They span from disease prevention via vaccination, strategies treating the host or the host microbiota, or modulation of the pathogenicity of microbes to antimicrobials by single active or combinations. *FMT = fecal microbiota transplantation. **Resistance inhibitors are lactamase-inhibitors, permeabilizers, or efflux-pump inhibitors

Does this not mainly concern sanitation councils and drug discovery research? Is this actually a topic for drug delivery scientists? Undoubtedly, strategies for better disease prevention and responsible use of anti-infectives are needed along with the discovery and development of novel active compounds. However, research for better anti-infective medicines also deserves to be a topic in drug delivery. Figure 2 provides an overview of anti-infective strategies now clearly going beyond classical antibiotic small molecule therapies. The diversity of actives used in such therapeutic concepts can profit from various delivery systems and formulations. Drug delivery can enable safer vaccines based on proteins or nucleotides. This will enable vaccination against pathogens so far not suitable or highly complicated for vaccination by killed or attenuated microorganisms because of their high genetic variability, the spread of multiple serogroups/types, or complications by preexisting immune responses for similar viral families (like HIV or dengue virus). Reverse vaccinology can identify optimized epitope display by broad HLA groups, and biotechnology allows engineering of immunogens and production of glycoproteins and construction of epitope-optimized proteins, or nucleotide sequences encoding them (pDNA, mRNA, or self-replicating RNAs). The key to vaccination is the delivery of antigens with optimized antigen presentation to achieve a sufficient broad protective immune response [7]. Thus, the combined efforts of understanding the disease (pathogen life cycle and immune response), bioinformatics, biotechnology, and delivery can be optimized through existing “platform technologies” for vaccine development including mRNA-based, virus-like-particle-based, and viral-vector-based vaccines. The speed in SARS-CoV-2 vaccine development was due to the investment in such platform technologies before the emergence of the virus and the already outlined plans for regulatory approval plans in a pandemic emergency. Such vaccine platform technologies, no matter whether viral- or non-viral delivery, predetermine the immune response type and safety considerations and allow alignment of manufacturing procedures. A technology that allows a broad range of antigens to be engineered with moderate technology adaptation such as the mRNA platform can be a game changer in the development effort for vaccines. The non-profit research organization IAVI with a long tradition in vaccination recently announced the first results of their clinical trial of an HIV vaccine in phase 1 [8]. The key to this success was the identification of broadly neutralizing antibodies, targeting a specific B lymphocyte population, and the use of delivery platforms (currently replicating not only viral vector vesicular stomatitis virus but also mRNA) [9].

Prevention of infections reduces the need for antibiotics, a strategy that is not limited to humans, but also can decrease antibiotic use in animals. Reducing the use of antibiotics in animal housing and food production is an essential piece of the strategy against AMR. A sufficiently high concentration of anti-infectives is essential to be effective and prevent the development of drug resistance. Drug delivery can ensure delivery of the right dose to the target at the right time for as long as is necessary. Optimizing bioavailability and pharmacokinetics by the dosage form is a classical theme in drug delivery and may contribute a lot to minimizing the risk of resistance development. Targeted delivery strategies could open the therapeutic window for drugs previously considered too toxic for systemic delivery (e.g., colistin in some pulmonary applications [10, 11]. Delivery strategies/systems, ensuring effective doses, and reducing adverse effects are needed. Improved delivery and synergistic combinations might increase the time we can use existing antibiotics, an important issue given the challenges in the discovery of novel antibiotics. Gained knowledge on the pathogen mechanisms (e.g., biofilm communication, efflux pumps, molecules interfering with the host immune response) of certain bacteria of high antibiotic-resistance tendency that can be used as anti-infective targets. Some modern anti-infectives, like e.g., quorum-sensing inhibitors, do not aim for killing to reduce evolutionary pressure and therefore are referred to better as pathoblockers rather than antibiotics. However, because they must reach intracellular targets, their bioavailability at the site of action is much limited by the permeability across the bacterial cell envelope (“cell wall”) especially in case of gram-negatives [12].

While research on advanced delivery technologies has concentrated for a long time on oncology, the last few years saw an increase in delivery approaches to improve anti-infective therapies. Some were mainly transferring developed particle technology to other diseases, but sophisticated approaches for highly tailored systems using bacterial enzymes to trigger drug release, mimicking pathogen entry paths, or interfering with biofilm growth mode have also been developed. Achieving selectivity in antimicrobials might be supported by carrier systems. Attempts to use nucleotides as anti-infectives with the possibility of high selectivity to kill or modify bacteria are promising new concepts, but it need strategies for delivery into bacterial cells. Host-directed therapies, e.g., to limit cytokine storms and tissue damage in viral infections may be realized by delivery strategies for biomacromolecules. Non-invasive antibody delivery at mucosal barriers as a preventive strategy is suggested. There are many possibilities for delivery technologies, and several examples are reported in this special issue, also covering quite diverse approaches. Also preferred by most drug researchers, animal models are not the best choice — regardless of ethical aspects — since infection processes are species-specific (by the microbial adaption to the environment and the immune response). Thus, more research efforts are needed to develop and validate alternative methods, preferably based on human cells and tissues, and implementing (patho)physiology-based pharmacokinetics to investigate interrelations between microbe, human and therapy either in vitro or in silico [13].

Drug delivery is able to contribute to our overall attempts for combatting and preventing the various threats associated with infectious diseases. Currently, many of these approaches are in academia and funded by public resources. Clinical translation of promising concepts is a financial challenge impossible for academia without potent partners. It is important to have such translational aspects in view on time (e.g., securing intellectual property, planning technological approaches  like scale up or biocompatibility of novel excipients early). Further, it is pertinent to develop models for selecting and propagating the most promising anti-infectives. Institutions, networks, and strategy plans were created like GARD-P (https://gardp.org/), Carb-X (https://carb-x.org/), and BEAM Alliance (https://beam-alliance.eu/) to foster antibiotic drug discovery and development. Historically, drug delivery is often seen as a part of “development” rather than as “research”. Targeted delivery and transport of drugs across biological barriers require an advanced understanding of the relevant biological and physicochemical fundamentals. Like building an airplane or a spacecraft must overcome the laws of gravity, advanced drug delivery goes beyond the mere application of well-established principles. The development of novel anti-infectives makes it necessary to spend money and time early on to ensure optimized delivery and accurate dosing are achieved (otherwise risking a short time before resistance development).

Challenges always come with opportunities. The current awareness of the threats of infectious disease and their impact on modern life may provide an opportunity to rethink our priorities and lead to developing contingency interventions with greater engagement. Drug delivery will play an important role in these interventions. In this spirit, we hope you enjoy the science of this special issue collection where Eastern and Western drug delivery approaches to the relentless challenge of infectious disease worldwide are highlighted.

In the Western perspective, the issue offers a collection of thirteen experimental or review articles. For example, exciting developments are discussed by researchers at the University of Copenhagen on emerging nanoparticle pulmonary delivery systems to treat respiratory tract bacterial infections [14]. Thiyagarajan et al. enhance the pulmonary delivery potential of antimycobacterial nanopharmaceuticals by spray-drying the nanoparticles to form lactose-leucine microparticles with potentially more favorable aerodynamic properties [15]. Along the same delivery route, the challenge of delivering large anti-infectious biologics, antibodies, is presented by Mayor et al. [16] while Juntke et al. present a novel model of human bronchial epithelial cells cultivated at the air–liquid interface (ALI) and infected with a *Pseudomonas aeruginosa* biofilm that could have a large impact on the testing of such aerosolized anti-infective nanoparticle delivery systems [17]. The use of lipid delivery systems and their potential to ameliorate antimicrobial therapeutic effects are expounded in Clive Prestidge’s “Nano-fats for bugs” review [18] and highlighted again by Ryan et al. exploration of solid lipid nanoparticles for delivery of dual-acting antimicrobial peptides [19]. Nnamani et al. also investigate lipid-based nanogels for the delivery of artemether to treat malaria in this issue [20]. Across several of the publications, and indeed in Albayaty et al. work on fighting infections caused by fungal biofilms [21] and Re et al. review on treating recurrent candidiasis [22], nanotechnology and drug delivery strategies, in the form of lipid or polymeric nanoparticles and micelles, show large potential in defeating biofilms of resistant strains. Mucoadhesive polymers are also highlighted by Senel et al. to treat fungal infections in the mouth [23]. We are reminded of the challenges of treating infection in hospital settings in two reviews in this issue, from researchers at John Hopkins University in the USA and at Goethe University Frankfurt, on emerging strategies of local drug delivery to combat post-operative infections from implantable biomaterials [24] and on the treatment of infected wounds respectively [25]. Lastly, from the Western perspective, given the focus on vaccine production during the recent pandemic, the issue would not be complete without an article highlighting the importance of nanotechnology and drug delivery in vaccine development. Cordeiro et al. work reports on their novel polysaccharide nanoparticles with immunomodulatory properties, highlighting their potential in future vaccine development [26].

In the Eastern perspective, the issue offers a collection of fifteen relevant research or review papers, contributed by world-leading researchers. For example, various biomaterials have been exploited to exhibit unique functions and promote innovations in the field of drug delivery. Meng et al. review the applications of chitosan-based systems for the treatment of infectious diseases, as well as the design considerations including adhesive ability, controlled release function, and other physical properties [27]. Skin application of chitosan-based hydrogels was reported by Huang et al. [28]. Chitosan-based hydrogels showed improved recovery of hydrofluoric acid burns with the prevention of skin infections. These topical hydrogels can be useful for the versatile delivery of antibiotics and antiviral drugs for the prevention of secondary infections in skin injury. Prabhu et al. studied injectable mannose-modified chitosan nanoparticles for enhanced intracellular delivery of rifampicin to the sites of tuberculosis [29], which has been one of the major infectious diseases threatening public health in Asia. Due to the intracellular survival pathogenicity of Mycobacterium tuberculosis, effective delivery systems could be essential. Chogale et al. focused on the development of the dry powder inhaler formulation for the treatment of tuberculosis [30].

Stimuli-responsive materials have been shown to achieve highly efficient drug delivery and targeted treatment. Wei et al. present the ultrasound-responsive polymer-based drug delivery systems for biomedical applications with introduction of the stimulation mechanism and typical formulations, including polymeric nanodroplets, micelles, microbubbles, and hydrogels [31]. Deng and Liu discuss the potential of utilizing inflammation-associated pathological milieu, such as oxidative stress, acidic pH, and overexpressed enzymes to trigger the release of therapeutic agents from responsive drug carriers for improved treatment and management of chronic inflammatory diseases [32].

Nanotechnology has been extensively explored for bioapplications including drug delivery and tissue engineering. Yang et al. highlight the recent advances in the development of therapeutic nanoformulations against various infectious for pathogen- and host-targeted antiviral delivery [33]. Fan et al. give an overview of antimicrobial nanomedicine ocular infections including keratitis and endophthalmitis [34]. DNA-derived nanostructures that selectively capture gram-positive bacteria were reviewed by Kim et al. [35]. Besides, Wang et al. survey the application of titanium dioxide nanotubes as drug carriers for infection control and osteogenesis of bone implants [36]. Cai et al. review the nanotechnology for physical sterilization focusing on topographical nanostructures [37].

Especially with the pandemics of COVID-19, vaccine delivery systems have received unprecedented high attention for the public health of the globe. Vaccine delivery systems for prevention of infectious diseases are overviewed by Kim et al. [38]. This review covers lipid and polymer-based non-viral delivery technologies for recently emerged viral infectious diseases including COVID-19 and Zika virus. In addition, transdermal drug delivery systems such as patches and microneedles have emerged as an alternative administration method with minimal invasion and less pain. Wang et al. describe transdermal delivery of antiviral drugs and vaccine antigens against COVID-19, influenza, and herpes simplex [39]. As transdermal delivery systems, the merits and limitations of transdermal patches and microneedles have been discussed. Oh et al. describe a microneedle-assisted vaccination for immunization through the buccal mucosa, which demonstrates a high serum IgG titer comparing to traditional transmucosal delivery [40].

The exploration of infectious disease detection has also attracted tremendous attention especially during the ongoing COVID-19 pandemic. Zhang et al. summarize the recent advances in self-luminescent systems for the detection of bacteria, fungi, and viruses byusing the advantages of ATP-derived self-luminescence as the light source [41]. In addition, they also provide an overview of self-luminescence system-based photodynamic therapy for treating infectious diseases.

Collectively, this special issue highlights the diverse research work and most advanced progress in the field of prevention and treatment of infectious diseases. Although several hurdles may remain for translation to clinical trials, the experimental delivery technologies presented could provide a dynamic platform for future pipelines of new drugs fighting against infectious diseases. We also hope that these important endeavors would provide the opportunity for the development and translation of drug delivery systems to fight infectious diseases, especially for COVID-19.
